# The mitogenome of a 35,000-year-old *Homo sapiens* from Europe supports a Palaeolithic back-migration to Africa

**DOI:** 10.1038/srep25501

**Published:** 2016-05-19

**Authors:** M. Hervella, E. M. Svensson, A. Alberdi, T. Günther, N. Izagirre, A. R. Munters, S. Alonso, M. Ioana, F. Ridiche, A. Soficaru, M. Jakobsson, M. G. Netea, C. de-la-Rua

**Affiliations:** 1 Department of Genetics, Physical Anthropology and Animal Physiology, University of the Basque Country (UPV/EHU), Barrio Sarriena s/n. 48940 Leioa, Bizkaia, Spain; 2 Department of Organismal Biology, Uppsala University, 75236 Uppsala, Sweden; 3 Natural History Museum of Denmark, University of Copenhagen, Øster Voldgade 5-7, 1350 Copenhagen, Denmark; 4 Human Genomics Laboratory, University of Medicine and Pharmacy of Craiova, Bvd. 1 Mai no 66, Romania; 5 Department of Internal Medicine and Radboud Center for Infectious Diseases, Radboud University Nijmegen Medical Centre, Nijmegen, The Netherlands; 6 Museum of Oltenia, History and Archaeology Department, Madona Dudu str. no. 14, Craiova, Romania; 7 “Fr. J. Rainer” Institute of Anthropology, Romanian Academy, Eroii Sanitari 8, P. O. Box 35-13, Romania; 8 Science for Life laboratory, Uppsala University, 75123 Uppsala, Sweden

## Abstract

After the dispersal of modern humans (*Homo sapiens*) *Out of Africa*, hominins with a similar morphology to that of present-day humans initiated the gradual demographic expansion into Eurasia. The mitogenome (33-fold coverage) of the Peştera Muierii 1 individual (PM1) from Romania (35 ky cal BP) we present in this article corresponds fully to *Homo sapiens*, whilst exhibiting a mosaic of morphological features related to both modern humans and Neandertals. We have identified the PM1 mitogenome as a basal haplogroup U6*, not previously found in any ancient or present-day humans. The derived U6 haplotypes are predominantly found in present-day North-Western African populations. Concomitantly, those found in Europe have been attributed to recent gene-flow from North Africa. The presence of the basal haplogroup U6* in South East Europe (Romania) at 35 ky BP confirms a Eurasian origin of the U6 mitochondrial lineage. Consequently, we propose that the PM1 lineage is an offshoot to South East Europe that can be traced to the Early Upper Paleolithic back migration from Western Asia to North Africa, during which the U6 lineage diversified, until the emergence of the present-day U6 African lineages.

After the dispersal of modern humans *Out of Africa*, around 50–70 ky cal BP^1^,^2^,^3^,^4^ or earlier based on fossil evidence^5^, hominins with similar morphology to present-day humans appeared in the Western Eurasian fossil record around 45–40 ky cal BP, initiating the demographic transition from ancient human occupation (Neandertals) to modern human (*Homo sapiens*) expansion on to the continent^1^. The first insights of the genetics of early Eurasian modern humans were recently provided by four ancient human genomes: Ust’-Ishim (Western Siberia, 45 ky cal BP)^6^, Kostenki (Russia, 39–36 ky cal BP)^7^, Fumane 2 (Italy, 41–39 ky cal BP)^8^ and Peştera cu Oase (Romania, 37–42 ky cal BP)^9^. Population genetic analyses of modern-day human mitochondrial haplogroup distributions suggest that in conjunction with the Eurasian expansion, some populations initiated a back-migration to North Africa^10^,^11^,^12^,^13^. Although the first genome of an ancient African individual (Ethiopia, 4.5 ky cal BP) identified a back-migration from Eurasia to Africa within the last 4.500 years^14^, the scarcity of older human remains in North Africa has prevented researchers from obtaining direct evidence of such a migratory phenomenon during the Paleolithic period. We present the mitochondrial genome (mitogenome) of the Peştera Muierii 1 (PM1) remains from Romania, directly dated to 35 ky cal BP^15^, which sheds new light on the Early Upper Paleolithic (EUP) migrations in Eurasia and North Africa.

We extracted DNA from two teeth and built 10 libraries from 3 DNA extracts, which were sequenced on an Illumina HiSeq 2500 platform (details in Supplementary Information). DNA fragments were aligned to the human mitochondrial genome, yielding an average coverage of 33×. This study was performed in accordance with biosafety guidelines regulation of the University of the Basque Country (UPV/EHU) and all experimental protocols were approved by the UPV/EHU. The sample was transferred with informed consent of the archeologists.

The fragmentation and nucleotide misincorporation patterns were consistent with a pattern of DNA damage typical of ancient DNA^16^ (Supplementary Figs 1 and 2). We used two methods to estimate contamination by checking the differences between individual reads and the consensus sequence. We first looked at conflicting alleles at nearly private sites in our sample (allele frequency <5% in 311 worldwide mitochondrial genomes)^17^. We observed two such sites in our sample, and only one out of 77 reads covering these sites showed a conflicting base, which corresponds to a contamination estimate of 1.3% (95% confidence interval: 0–3.8). Secondly, we applied contamMix^2^ which gives a Bayesian estimate of contamination based on mapping all reads against the consensus sequence as well as 311 other mitochondrial genomes. The Bayesian contamination estimate of 1.1% is similar to the estimate obtained using the first method.

We estimated the phylogenetic position of PM1 using Bayesian inference in a two-step analysis. First, we aligned the reconstructed mtDNA sequence with 10 other ancient mitogenomes, including two Denisovans^18^, two Neandertals^19^ and 6 ancient *Homo sapiens* from the EUP^2^,^6^,^7^,^9^ (Fig. 1A and Supplementary Table 2). The tree fully supports the position of PM1 within the modern *Homo sapiens* clade (Fig. 1A). None of the 63 ‘diagnostic’ positions (at which ten Neandertal mitogenomes differ from 311 present-day humans) appeared in PM1^19^,^20^,^21^,^22^,^23^,^24^. This observation is compelling as the morphology of PM1 exhibits features related both to modern humans and Neandertals^15^. Furthermore, the PM1 remains are not associated with any particular cultural techno-complex, as the lithic artifacts found at the site were related both to Mousterian (associated with Neandertals) as well as to Aurignacian assemblages (associated with early *Homo sapiens*)^25^. None of the reported mtDNA sequences from early modern humans have displayed Neandertal mitochondrial genomes^4^,^6^,^7^,^8^,^9^, although a low level of admixture has been detected in the nuclear DNA of modern humans^24^,^26^ and at higher proportions in one Paleolithic human^9^. As a second step, we estimated the mitogenomic position of PM1 within modern humans by analyzing 144 modern^27^ and 47 ancient human mitogenomes covering the known mitogenomic variability (Supplementary Tables 2 and 3). The haplogroup of PM1 falls within the U clade (Fig. 1B and Supplementary Table 3), which derived from the macro-haplogroup N possibly connected to the *Out of Africa* migration around 60–70 ky cal BP^1^,^2^,^3^,^4^. In line with this, the Peştera cu Oase individual that lived on the current territory of Romania, albeit slightly earlier than PM1 (37–42 ky cal BP) also displays haplogroup N^9^.

The analysis of the PM1 mitogenome polymorphisms revealed 15 nucleotide changes with respect to the rCRS^28^, identifying the PM1 mitogenome as a basal haplogroup U6* (Supplementary Table 1). One of these polymorphisms is a private mutation, T10517A, not previously found in any mitochondrial genome. The U6 haplogroup is the only sub-haplogroup within the U clade currently present in Africa, showing an increasing frequency gradient from Eastern (1.09–1.57% in Egypt) to Western North Africa (8.89% in the Magreb). A similar longitudinal gradient is present in the Southern European populations (from 0.19% in Eastern Mediterranean to 1.12% in South Spain)^29^,^30^ (Fig. 2B). The U6 haplotypes found in present-day Europeans have been attributed to African sources, mainly to the historic Moorish expansion, but also to prehistoric influence since Neolithic times^29^,^30^. Hence, PM1 is the first basal U6 haplogroup found in Europe that is not connected to recent migration from Africa.

The mitogenome from PM1 offers important information in order to understand human population movements during the Paleolithic Age related to the haplogroup U6. While all the extant U6 haplotypes belong to derived branches, i.e. U6a’b’d (characterized by transition, 16219) or to the less frequent U6c (characterized by a set of eleven mutations, 150, 437, 793, 3688, 4965, 5081, 11013, 13879, 15244, 16169, 16189)^30^ (Fig. 2A), the haplotype of the PM1 individual belongs to the basal U6 haplogroup from which the rest of haplotypes were derived (Fig. 2A). This scenario confirms that the U6 mitochondrial lineage has a Eurasian origin, supporting the hypothesis of an early back-migration from Eurasia to North Africa in the EUP^10^,^11^,^30^.

Individuals carrying haplogroup U possibly spread westward from Western Asia around 39–52 ky, reaching Europe as signaled by haplogroup U5, and North Africa signaled by haplogroup U6, which likely represents a genetic signal of a EUP return of *Homo sapiens* from Eurasia to North Africa^11^,^29^,^30^. The time of the most recent common ancestor (TMRCA) for U6 was estimated to 35.3 (24.6–46.4) ky BP^29^,^30^. Thus it has been proposed that the lineage originated somewhere in Western Asia^11^,^29^,^30^. We found a basal U6 in South East Europe, on the current territory of Romania 35 ky BP, suggesting that either the U6 lineage originated in Eastern Europe or the TMRCA of U6 is older than 35 ky. Our estimates of the haplogroup U6 TMRCA that incorporate ancient genomes (including PM1) set the formation of the U6 lineage back to 49.6 ky BP (95% HPD: 42–58 ky) (using a mutation rate of 2.06* 10^−8^ SD = 1.94 * 10^−9^) (Fig. 1). Our estimates are almost identical in age to that by reference^11^ (45.1 ± 6.9 ky). Given the presence of a basal U6 mitogenome in Romania 35 ky BP, the distance between Western Asia and Romania, and the estimated diffusion pace of hunter-gatherer populations^30^ suggest that the early populations carrying haplogroup U6 most likely started their spread to Eastern Europe before 40 ky BP.

It is unclear whether the haplogroup U6 diversified in Africa or arrived to the continent as an already diversified lineage. However, the detection in South East Europe (Romania) of a basal U6* haplotype presenting only two of the diagnostic mutations (3348 and 16172) of modern-day U6 haplogroups (Fig. 2A and Supplementary Table 3) strongly points to an “on route” differentiation of U undifferentiated lineages to basal U6 lineages before reaching Africa.

Considering the mitogenome of PM1, we suggest that the PM1 lineage could be an offshoot to South-East Europe of the EUP migration that lead U6 from Western Asia to Africa during which it diversified until the emergence of the present-day U6 African lineages. Although nuclear sequence data are needed to clarify the genetic relationship of PM1 to modern-day and archaic humans, the mitogenome establishes a link between PM1 and the ancestor of the U6 haplogroup in Eurasia.

## Additional Information

**How to cite this article**: Hervella, M. *et al*. The mitogenome of a 35,000-year-old *Homo sapiens* from Europe supports a Palaeolithic back-migration to Africa. *Sci. Rep*. **6**, 25501; doi: 10.1038/srep25501 (2016).

## Supplementary Material

Supplementary Information

## Figures and Tables

**Figure 1:**
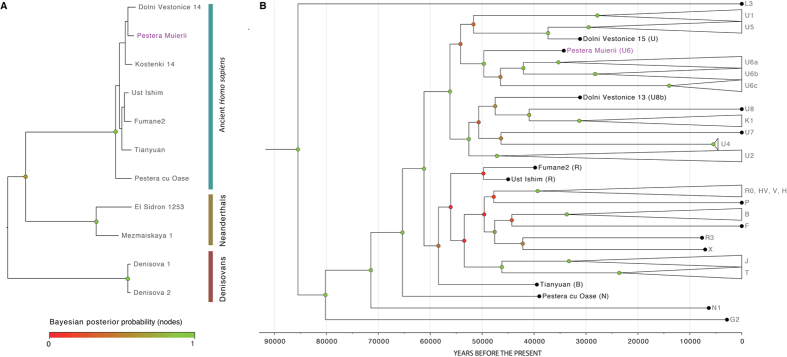
Phylogenetic analyses of the Peştera Muierii-1 (PM1) mitogenome (35 Kcal BP, Romania). (**A**) Unconstrained Bayesian phylogenetic analysis including ancient *H. sapiens*, Neandertals and Denisovans. (**B**) Unconstrained Bayesian phylogenetic analysis including ancient and present-day *H. sapiens*. The tree is time-calibrated using node ages. The color of node dots indicates the posterior probability (pp): green dots = maximum robustness, yellow dots = slight robustness, red dots = low robustness.

**Figure 2:**
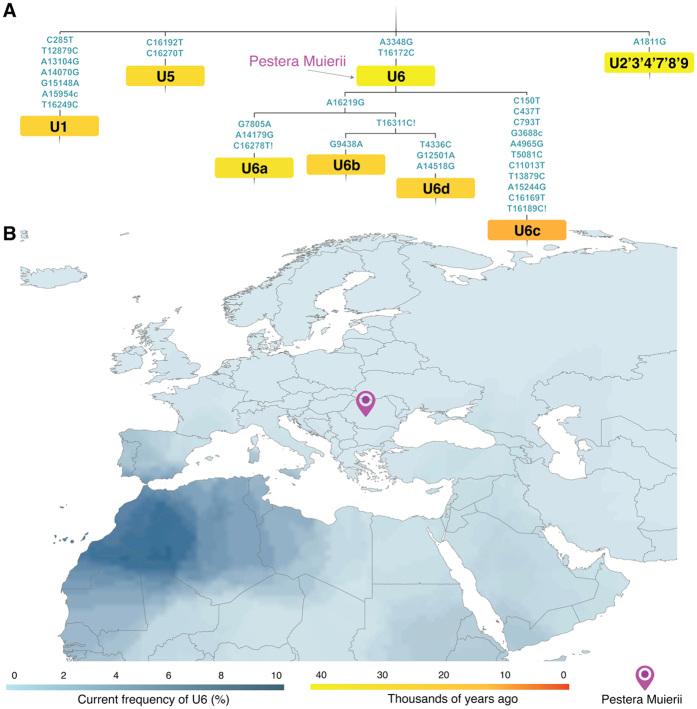
Distribution of the U6 mitochondrial lineages. (**A**) Phylogenetic analysis and temporal estimates for lineages including the Peştera Muierii-1 (PM1) from the mitochondrial tree. (**B**) Location of the Peştera Muierii cave and surface map based on current frequencies of U6 lineages^30^; the European borders map was generated in ArcMap 10.1 (ESRI, http://www.esri.com) by modifying the World Borders Dataset (http://www.thematicmapping.org/downloads/world_borders.php), which is licensed under the Attribution-ShareAlike 3.0 Unported license. The license terms can be found on the following link: http://creativecommons.org/licenses/by-sa/3.0/ (This map was created by A.A.).
