# Supplementary material for: The mitogenome of a 35,000-year-old Homo sapiens from Europe supports a Palaeolithic back-migration to Africa
Source: Sci Rep. 2016 May 19;6:25501. doi: 10.1038/srep25501 (PMC4872530; doi:10.1038/srep25501)
Supplement: Supplementary Information [file srep25501-s1.doc]

**Supplementary Materials for ‘The mitogenome of a 35,000-year-old *Homo sapiens* from Europe supports a Palaeolithic back-migration to Africa’**

M. Hervella^1^, E.M. Svensson^2^, A. Alberdi^3^, T. Günther^2^, N. Izagirre^1^, A.R. Munters^2^, S. Alonso^1^, M. Ioana^4, 5^, F. Ridiche^6^, A. Soficaru^7^, M. Jakobsson^2, 8^, M.G. Netea^5^& C. de-la-Rua^1*^

^1^ Department of Genetics, Physical Anthropology and Animal Physiology. University of the Basque Country UPV/EHU, Barrio Sarriena s/n. 48940 Leioa, Bizkaia, Spain.

^2^ Department of Organismal Biology, Uppsala University, 75236 Uppsala, Sweden.

^3^ Natural History Museum of Denmark, University of Copenhagen, Øster Voldgade 5-7, 1350 Copenhagen , Denmark.

^4^ Human Genomics Laboratory, University of Medicine and Pharmacy of Craiova, Bvd. 1 Mai no 66, Romania.

^5^ Department of Internal Medicine and Radboud Center for Infectious Diseases, Radboud University Nijmegen Medical Centre, Nijmegen, The Netherlands.

^6^ Museum of Oltenia. History and Archaeology Department, Madona Dudu str. no. 14, Craiova, Romania.

^7^ Fr J. Rainer” Institute of Anthropology, Romanian Academy, Eroii Sanitari 8, P. O. Box 35-13.

^8^ Science for Life laboratory, Uppsala University, 75123 Uppsala, Sweden.

***** Correspondence author: Concepción de-la-Rúa, Dept. of Genetics, Physical Anthropology and Animal Physiology. University of the Basque Country UPV/EHU, Barrio Sarriena s/n. 48940 Leioa, Bizkaia, Spain. [conchi.delarua@ehu.eus](mailto:conchi.delarua@ehu.eus)

1. **MORPHOLOGICAL, CHRONOLOGICAL AND CULTURAL CONTEXT OF PESTERA MUIERRI REMAINS**

The human remains from the Peştera Muierii (Cave of the Old Woman) were discovered in 1952 near Baia de Fier, Gorj County, Romania, in a multi-chambered karstic system *^1^.* Human remains from 3 individuals (Peştera Muierii 1, Peştera Muierii 2 and Peştera Muierii 3) were found in this cave ^2^. The human remains from the Peştera Muierii-1 (PM1) were directly dated to 30,000 radiocarbon years before present (30 ka 14C BP), corresponding to 35 ka cal BP (calibrated age based on CalPal 2005) ^1^*.*

When compared to other Late Pleistocene samples, early and roughly contemporary humans and Neandertals, as well as modern-day humans ^1, 3-4^ most of the morphological traits of PM1 fall within the range of modern-day humans (based on e.g. the morphology of the zygomatic bone and nasal floor). Yet some parts of the facial shape and dentition wear are within the range of variation found in Neandertals(e.g. the prominent occipital bun, and large interorbital breadth) ^1^*.* In its heavily worn dentition PM1 exhibits Neandertal patterns:

The I2 has the remains of marginal ridges but no evidence of a lingual tubercle, indicating some shoveling ^5^*.*

The C1 is featureless, and the subrectangular M1s seem to lack the metacone reduction and hypocone expansion ^6^.

The M2s seem to have had some hypocone reduction, and the right M3 is a peg tooth, a feature occasionally found among Neandertals and middle Upper Paleolithic modern humans ^7^

Thus, the PM1 exhibits a mosaic morphology composed of modern traits as well as a number of archaic and/or Neandertal features, which has been interpreted as the result of the complex dynamics of human reproductive patterns when modern humans dispersed westward across Europe during the Late Pleistocene ^1^.

In the South-East Europe region encompassing the current territory of Romania, the interval of time between 34–26 ky BP is the transitional period from the Middle Paleolithic to the Early Upper Paleolithic. The PM1 remains (35 ky cal BP) were not associated to a certain cultural techno-complex, but were found with lithic artifacts related both to Mousterian assemblages (associated to Neandertals) and to Aurignacian assemblages (associated to early modern humans) ^2^.

1. **DNA ISOLATION IN BILBAO**

DNA was extracted from the upper right 3^rd^ molar and the upper left 2^nd^ molar belonging to PM1 using a phenol/chloroform protocol ^8^. The teeth did not show signs of caries or deep fissures that might extend into the dental pulp.

All pre-sequencing steps were performed in a sterile chamber with positive pressure, free of modern DNA, in which no post-PCR process had ever been carried out. Ancient DNA results were validated through the application of standard aDNA authentication criteria ^9-10^*.* Real-time quantitative PCR (RT-qPCR) was performed to quantify the number of molecules of mtDNA in the extracts, this ranged from 7368-2354 mol/µl ^8^*.* To detect post-mortem damage six PCR products of HVR-I were also cloned using TOPO TA Cloning® Kits (Invitrogen), we found 14.5 changes/PCR product cloned.

1. **LIBRARY BUILDING AND SEQUENCING IN UPPSALA**

In total ten double stranded libraries for Illumina sequencing were built from 20 μl of extract using the protocol by ^11^ with modifications as in ^12^ qPCR was used to assess the optimal number of PCR cycles for amplification. Indexing PCRs were set up in a total volume of 25µl, using AmpliTaq GOLD DNA polymerase (Thermo Fisher). Each library was amplified in quadruplicates with an index in the P7 primer, PCR products from the same library were pooled and cleaned with Agencourt AMPure XP beads (Beckman Coulter) and quantified on a TapeStation (Agilent Technologies). Cleaned libraries were pooled in equimolar concentration and deep sequenced on an Illumina HiSeq 2500 using v4 chemistry at NGI platform at Uppsala University.

**4. SEQUENCE PROCESSING AND MAPPING**

We trimmed adapters and merged paired-end reads (requiring an overlap of at least 11bp). The merged sequences were then mapped to the Revised Cambridge Reference Sequence (rCRS) ^13^ as well as the Reconstructed Sapiens Reference Sequence (RSRS) ^14^ mitochondrial reference sequences using BWA ^15^ with seeding disabled using the non-default parameters *-o* 2 and *-n* 0.04. If several fragments mapped to identical start and end coordinates, we considered them as PCR duplicates and collapsed them into consensus sequences. Less than 10% mismatching positions were required between the sequences and the reference genome. Furthermore, we discarded fragments shorter than 35 bp (Supplementary Figure S1). To call a consensus sequence for the mitochondrial genome of PM1, we used *mpileup* and *vcfutils* provided by samtools ^16^ requiring mapping and base qualities of at least 30. Scripts provided by (doi:10.1007/978-1-61779-516-9_23, <https://bioinf.eva.mpg.de/fastqProcessing/>) to process the NGS data.

**5. ESTIMATES OF MITOCHONDRIAL CONTAMINATION AND aDNA AUTHENTICATION**

We considered private or nearly-private (allele frequency of less than 5% in 311 modern mitochondrial genomes) alleles in the PM1 consensus sequence to estimate contamination ^17^. Only reads with a mapping quality of ≥30 and sites with base call quality of ≥30 were considered. We restricted the analysis to sites with a minimum coverage of 10 and excluded sites were the consensus allele was C or G at a transition sites in order to avoid inflated estimates due to post-mortem damage. We obtained a point estimate for the mitochondrial contamination by dividing the number of non-consensus alleles by the number of all reads covering informative sites. The 95% confidence interval for the contamination was derived from a binomial approximation.

To further authenticate the results DNA fragmentation and nucleotide misincorporation patterns in the PM1 sample reads were done by using custom scripts. The PM1 reads show the for aDNA typical increased levels cytosine to thymine and guanine to adenine nucleotide at the 5'-end and 3'- termini of sequences, respectively (Supplementary Figure S2) ^18-19^. Furthermore the fragment length distribution is also in the range typically of aDNA (Supplementary Figure S1).

**6. PHYLOGENETIC ANALYSES**

Two Bayesian phylogenetic analyses were performed to infer the phylogenetic position of PM1. In both analyses, the best-fit model of evolution was selected using jModeltest 2 ^20^ under AIC, BIC, and AICc criteria prior to Bayesian analyses. Bayesian analyses were carried out using BEAST 2 ^21^. Two simultaneous runs of 50 million generations were conducted for the datasets and trees were sampled every 1,000 generations, with the first 25% discarded as burn-in. Samples from the posterior were checked for acceptable effective sample sizes (>200) and the adequate convergence of the MCMC chains was checked using Tracer 1.6 ^22^.

The first analysis aimed to ascertain the phylogenetic position of PM1 within the different hominins from Eurasia which lived during the Middle and Upper Paleolithic. The analysis included the complete mitochondrial genomes of 10 hominins found in Eurasia between 30 and 65 ky BP, including PM1, five modern *Homo sapiens*, two Neandertals and two Denisovans (Supplementary Table 3). The analysis was performed using the HKY+G+I substitution model, relaxed clock log-normal and Yule tree prior, indicating the tip dates of the samples.

Once the phylogenetic location of PM1 within modern humans had been established, we performed a second analysis using the complete mitogenomes of 190 modern *Homo sapiens*, including eight individuals from the Early Upper Paleolithic, one sample from the Late Upper Paleolithic, 37 humans from the Neolithic, one individual from the 15^th^ century and 143 modern samples (Supplementary Table S3). The modern humans were selected from all the individuals with published mitogenomes and covered the whole phylogenetic diversity within the N hg lineage, with special emphasis in the U lineage. The analysis was carried out using the HKY+G+I substitution model, strict molecular clock and coalescent constant population tree prior, indicating the tip dates of the samples.

**Supplementary Figure S1:** Fragment length distribution for all sequences used to call the consensus mitogenome (mapping to the mitochondrial reference with a minimum mapping quality of 30).


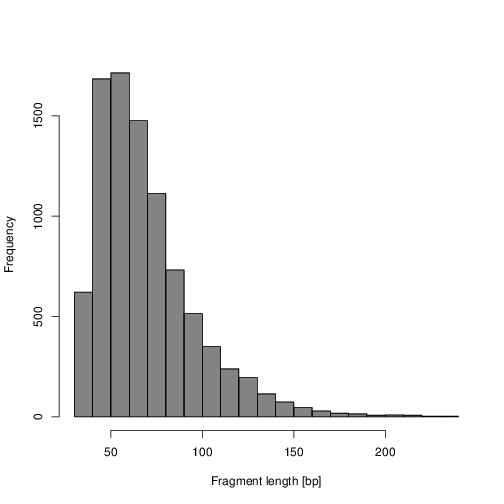


**Supplementary Figure S2:** Damage pattern for all sequences used to call the consensus mitogenome (mapping to the mitochondrial reference with a minimum mapping quality of 30).

**
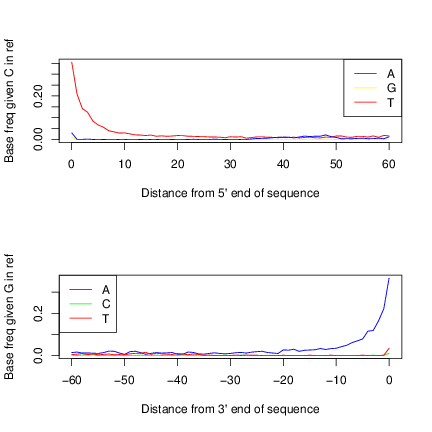
**

**Supplementary Table S1. All sites differing between the PM1 mitogenome and the rCRS^13^. Only reads with a minimum mapping quality of 30 and alleles with a base quality of 30 or more were considered. The column PM1 does not show the consensus allele, it shows any alternative allele found among all reads covering that site. The private mutations of rCRS at positions 4769 and 8860 lacked enough coverage, based on our quality criteria, in the PM1 mitogenome.**

| **SNP pos** | **rCRS** | **Alternative allele found in PM1 reads** | **Number of reads supporting the alternative allele** | **Total number of reads covering the site** | **%** |
| --- | --- | --- | --- | --- | --- |
| 73 | A | G | 28 | 28 | 100 |
| 263 | A | G | 66 | 66 | 100,0 |
| 310 | T | insC | 4 | 9 | 44,4 |
| 679 | C | T* | 2 | 12 | 16,7 |
| 750 | A | G | 32 | 34 | 94,1 |
| 1239 | C | T* | 3 | 19 | 15,8 |
| 1438 | A | G | 13 | 13 | 100,0 |
| 2496 | G | A* | 2 | 7 | 28,6 |
| 2706 | A | G | 50 | 53 | 94,3 |
| 3105 | A | delC | 1 | 37 | 2,7 |
| 3348 | A | G | 48 | 48 | 100,0 |
| 3566 | C | T* | 4 | 33 | 12,1 |
| 4850 | C | T* | 2 | 10 | 20,0 |
| 5294 | C | T* | 2 | 7 | 28,6 |
| 5300 | C | T* | 2 | 9 | 22,2 |
| 5312 | C | T* | 2 | 11 | 18,2 |
| 5871 | G | A* | 2 | 12 | 16,7 |
| 5881 | G | A* | 2 | 7 | 28,6 |
| 6651 | G | A* | 2 | 13 | 15,4 |
| 7028 | C | T | 4 | 4 | 100,0 |
| 7252 | G | A* | 4 | 31 | 12,9 |
| 7648 | C | T* | 2 | 11 | 18,2 |
| 8083 | C | T* | 2 | 13 | 15,4 |
| 8609 | C | T* | 2 | 6 | 33,3 |
| 9209 | G | A* | 2 | 5 | 40,0 |
| 9470 | C | T* | 2 | 7 | 28,6 |
| 10517 | T | A | 15 | 16 | 93,8 |
| 11467 | A | G | 39 | 40 | 97,5 |
| 11719 | G | A | 11 | 11 | 100,0 |
| 12308 | A | G | 45 | 45 | 100,0 |
| 12372 | G | A | 27 | 27 | 100,0 |
| 12838 | G | A* | 5 | 41 | 12,2 |
| 14766 | C | T | 30 | 31 | 96,8 |
| 15326 | A | G | 57 | 60 | 95,0 |
| 16172 | T | C | 27 | 30 | 90,0 |

* Sites where potential post-mortem damage could explain the non-consensus reads.

**Supplementary Table S2**. Mitogenomes used in phylogenetic analysis (Fig. 1A)

| **Sample** | **Taxonomy** | **Age (ky BP)** | **Genbank ref.** | **Reference** |
| --- | --- | --- | --- | --- |
| **Dolni Vestonice 14** | Modern human | 31 | KC521458 | 18 |
| **Kostenki 14** | Modern human | 38 | FN600416 | 23 |
| **Ust-Ishim** | Modern human | 45 | P[PRJEB6622](http://www.ebi.ac.uk/ena/data/view/PRJEB6622) | 24 |
| **Fumane 2** | Modern human | 40 |  | 25 |
| **Tianyuan** | Modern human | 39 | KC417443 | 23 |
| **El Sidron 1253** | Neanderthal | 39 | FM865409 | 26 |
| **Mezmaiskaya 1** | Neanderthal | 65 | FM865411 | 26 |
| **Denisova 1** | Denisovan | 41 | FN673705 | 27 |
| **Denisova 2** | Denisovan | 41 | FR695060 | 28 |

**Supplementary Table S3.** Ancient and present-day *Homo sapiens* mitogenomes used in phylogenetic analysis (Fig. 1B and Fig. 2A).

| **Ancient *H.sapiens*** | **Genbank/ENA reference** | **Date (y BP)** | **Reference** |
| --- | --- | --- | --- |
| **Dolni Vestonice 15** | KC521458 | 31155 | 18 |
| **Dolni Vestonice 13** | [KC521459](http://www.ncbi.nlm.nih.gov/entrez/query.fcgi?cmd=search&db=nucleotide&doptcmdl=genbank&term=KC521459) | 31155 | 18 |
| **La Braña** | JX186998. | 7827 | 29 |
| **NE1** | [SRP039766](http://www.ncbi.nlm.nih.gov/sra?term=SRP039766) | 7130 | 30 |
| **Bla14** | [KC521454](http://www.ncbi.nlm.nih.gov/entrez/query.fcgi?cmd=search&db=nucleotide&doptcmdl=genbank&term=KC521454) | 3604 | 18 |
| **Bla11** | [KC521454](http://www.ncbi.nlm.nih.gov/entrez/query.fcgi?cmd=search&db=nucleotide&doptcmdl=genbank&term=KC521454) | 3922 | 18 |
| **Loscbour** | [KC521455](http://www.ncbi.nlm.nih.gov/entrez/query.fcgi?cmd=search&db=nucleotide&doptcmdl=genbank&term=KC521455) | 8100 | 18 |
| **Oberkassel998** | [KC521457](http://www.ncbi.nlm.nih.gov/entrez/query.fcgi?cmd=search&db=nucleotide&doptcmdl=genbank&term=KC521457) | 14020 | 18 |
| **Bla20** | KF523407 | 8652 | 31 |
| **Motala2** | PRJEB6272 | 7900 | 32 |
| **Ire8** | [ERS434179](http://sra.dnanexus.com/samples/ERS434179) | 4579 | 33 |
| **Ajv70** | [ERS434179](http://sra.dnanexus.com/samples/ERS434179) | 4658 | 34 |
| **Kostenki 14** | [PRJEB7618](http://www.ebi.ac.uk/ena/data/view/PRJEB7618) | 37958 | 33 |
| **BR1** | [SRP039766](http://www.ncbi.nlm.nih.gov/sra?term=SRP039766) | 4050 | 30 |
| **BR2** | [SRP039766](http://www.ncbi.nlm.nih.gov/sra?term=SRP039766) | 3140 | 30 |
| **Pestera cu Ouase** | [PRJEB8987](http://www.ebi.ac.uk/ena/data/view/PRJEB8987) | 39000 | 24 |
| **Fumane2** |  | 39800 | 25 |
| **Ust-Ishim** | [PRJEB6622](http://www.ebi.ac.uk/ena/data/view/PRJEB6622) | 45000 | 35 |
| **Gok3** | [ERS434180](http://sra.dnanexus.com/samples/ERS434180) | 4921 | 33 |
| **Tianyuan** | [ERX181462](http://www.ncbi.nlm.nih.gov/sra/ERX181462) | 39475 | 18 |
| **BS11** | [KC521454](http://www.ncbi.nlm.nih.gov/entrez/query.fcgi?cmd=search&db=nucleotide&doptcmdl=genbank&term=KC521454) | 8180 | 18 |
| **HAL36** | [PRJEB8448](http://www.ebi.ac.uk/ena/data/view/PRJEB8448) | 7050 | 36 |
| **HAL32** | [PRJEB8448](http://www.ebi.ac.uk/ena/data/view/PRJEB8448) | 6145 | 36 |
| **SALZ18a** | [PRJEB8448](http://www.ebi.ac.uk/ena/data/view/PRJEB8448) | 6000 | 36 |
| **HQU4** | [PRJEB8448](http://www.ebi.ac.uk/ena/data/view/PRJEB8448) | 5700 | 36 |
| **SALZ57a** | [PRJEB8448](http://www.ebi.ac.uk/ena/data/view/PRJEB8448) | 5250 | 36 |
| **Iceman** | [ERP001144](http://www.ebi.ac.uk/ena/data/view/ERP001144) | 5228 | 37 |
| **Gok4** | [ERS434180](http://sra.dnanexus.com/samples/ERS434180) | 4921 | 33 |
| **TBD-BZH6** | [ERS434181](http://sra.dnanexus.com/samples/ERS434180) | 4500 | 33 |
| **BZH4** | KF523405 | 4350 | 31 |
| **BZH1** | KF523405 | 3800 | 31 |
| **Bla13** | KF523405 | 5463 | 31 |
| **QUEVIII4** | KF523405 | 3587 | 31 |
| **Bla13** | KF523405 | 3513 | 31 |
| **Bla10** | KF523403 | 3418 | 31 |
| **CroMagnon** | [KC521456](http://www.ncbi.nlm.nih.gov/entrez/query.fcgi?cmd=search&db=nucleotide&doptcmdl=genbank&term=KC521456) | 690 | 18 |
| **KO1** | [SRP039766](http://www.ncbi.nlm.nih.gov/sra?term=SRP039766) | 7650 | 30 |
| **KO2** | [SRP039766](http://www.ncbi.nlm.nih.gov/sra?term=SRP039766) | 7550 | 30 |
| **NE2** | [SRP039766](http://www.ncbi.nlm.nih.gov/sra?term=SRP039766) | 7100 | 30 |
| **NE3** | [SRP039766](http://www.ncbi.nlm.nih.gov/sra?term=SRP039766) | 7050 | 30 |
| **NE4** | [SRP039766](http://www.ncbi.nlm.nih.gov/sra?term=SRP039766) | 7100 | 30 |
| **NE5** | [SRP039766](http://www.ncbi.nlm.nih.gov/sra?term=SRP039766) | 7050 | 30 |
| **NE6** | [SRP039766](http://www.ncbi.nlm.nih.gov/sra?term=SRP039766) | 7100 | 30 |
| **NE7** | [SRP039766](http://www.ncbi.nlm.nih.gov/sra?term=SRP039766) | 6350 | 30 |
| **CO1** | [SRP039766](http://www.ncbi.nlm.nih.gov/sra?term=SRP039766) | 4750 | 30 |
| **IR1** | [SRP039766](http://www.ncbi.nlm.nih.gov/sra?term=SRP039766) | 2850 | 30 |

| **Present-day *H. sapiens from Phylotree data base ^38^***  **Genbank reference** | **Mitocondrial haplogroup** |
| --- | --- |
| **tztr024** | L3 |
| **JX273249** | K2b |
| **JX120741** | U6d |
| **JQ705429** | U5b2 |
| **HM852886** | K3 |
| **HM852844** | U1a3 |
| **HM852790** | U1a1 |
| **HM852789** | U1a2 |
| **HM043706** | K1a |
| **GU722602** | K1c1 |
| **FN600416** | U2 |
| **GU296570** | U5a1 |
| **EU330890** | U2b1 |
| **AY882393** | U8b1 |
| **AY882389** | U9a |
| **AY882379** | U2a1a |
| **AY275536** | U6a |
| **KC152578** | U6a8b |
| **KC152577** | U6a |
| **KC152576** | U6a2a2 |
| **KC152574** | U6a3d1a |
| **KC152569** | U6a2c |
| **KC152568** | U6c2 |
| **KC152567** | U6b1b |
| **KC152558** | U6a7b |
| **KC152555** | U6a7b |
| **KC152553** | U6a5a1 |
| **KC152552** | U6a8a |
| **KC152540** | U6a8b |
| **KC152539** | U6a8b |
| **KC152538** | U6a3a1a |
| **AY275537** | U6a7a1 |
| **AY275533** | U6a7a1a |
| **JX120774** | U6a2 |
| **JX120773** | U6a2 |
| **JX120772** | U6a2 |
| **JX120771** | U6a2 |
| **JX120770** | U6a2 |
| **JX120769** | U6a3 |
| **JX120768** | U6a4 |
| **JX120766** | U6a7a1a |
| **JX120765** | U6a7c1 |
| **JX120757** | U6a7a1a |
| **JX120756** | U6a7a1b |
| **JX120755** | U6a7a2 |
| **JX120754** | U6a7a2a |
| **JX120753** | U6a7a1 |
| **JX120752** | U6a7a1 |
| **JX120746** | U6d |
| **JX120745** | U6d1a |
| **JX120744** | U6d1 |
| **JX120743** | U6d3 |
| **JX120742** | U6d |
| **JX120740** | U6d3 |
| **JX120735** | U6b3a |
| **JX120734** | U6b3 |
| **JX120733** | U6b1 |
| **JX120732** | U6b1 |
| **JX120731** | U6a7b1 |
| **JX120725** | U6a6b |
| **JX120724** | U6a6b1 |
| **JX120723** | U6a5b |
| **JX120722** | U6a5b |
| **JX120721** | U6a5 |
| **JX120716** | U6a2b |
| **JX120715** | U6a2b |
| **JX120709** | U6a1b3 |
| **JX120708** | U6a3a2a |
| **JQ704030** | U6a3a2a |
| **JQ704008** | U6b2 |
| **JQ703902** | U6b3 |
| **JQ702816** | U6a1b2 |
| **JQ702612** | U6a1b3 |
| **JQ702118** | U6a7c |
| **JQ629405** | U6a8b |
| **HQ651713** | U6a3a |
| **HQ651712** | U6a3d1a |
| **HQ651711** | U6a3d1a |
| **HQ651710** | U6a3d1a |
| **HQ651709** | U6a3b0 |
| **HQ651703** | U6a3b1 |
| **HQ651702** | U6a2c |
| **HQ651701** | U6a1b3 |
| **HQ651700** | U6a2c3 |
| **HQ651693** | U6a1b1b |
| **HQ651692** | U6a1b1b |
| **HQ651691** | U6a1b1b |
| **HQ651690** | U6a1b1b |
| **HQ651689** | U6a1b2 |
| **HQ651680** | U6b1a |
| **HQ651679** | U6b1a |
| **HQ651678** | U6b1a |
| **HQ651677** | U6b1a |
| **HQ592783** | U6a5a1 |
| **HQ585390** | U6a3a1a |
| **HQ384209** | U6a2a1a |
| **HQ287880** | U6a1a1a |
| **HM775494** | U6c2 |
| **GU967378** | U6a7a1a |
| **GU433197** | U6a7a1b |
| **GU366066** | U6d1a |
| **FJ939330** | U6d1a1 |
| **FJ460539** | U6d1a2 |
| **EF064341** | U6d1b |
| **EF064340** | U6b1a1 |
| **EF064339** | U6a7b1 |
| **EF064338** | U6a7a1c |
| **EF064337** | U6a7a1b |
| **EF064336** | U6a6 |
| **EF064330** | U6a5a |
| **EF064329** | U6a5 |
| **EF064328** | U6a4 |
| **EF064327** | U6a4 |
| **EF064326** | U6a |
| **EF064325** | U6a |
| **EF064319** | U6a |
| **EF064318** | U6a |
| **EF064317** | U6a |
| **DQ856317** | U6d1a |
| **DQ523663** | U6d1a |
| **AY275529** | U6b1a0 |
| **AY275528** | U6b1a1 |

References

1. A. D. Soficaru *et al., Poc. Natl. Acad. Sci. U. S. A.* **103**. 17196-17201 (2006)

2. A. Doboş, *et al., ERAUL* **124**. Liège, 2010.

3. R. G. Franciscus *et al., J Hum Evol* **44**.701–729 (2003)

4. E. Trinkaus et al., *J Hum Evol* **45**. 245–253 (2003).

5. T. Crummett in *Aspects of Dental Biology: Palaeontology, Anthropology and Evolution*, 305–313 (1995).

6. S. E. Bailey, *J Hum Evol* **47**.183–198 (2004).

7. S. W. Hillson in *Early Modern Human Evolution in Central Europe* 179–223. (2005).

8. M. Hervella *et al*., Plos One **7.** e34417 (2012)

9. S. Paabo *et al., Annu Rev Genet* **38**. 645-679. (2004)

10 Gilbert and Willeslev*, Med Scold* **18**. 701–723 (2005).

11. M. Meyer and M. Kircher, *Cold Spring Harb Protoc.,* **6**. pdb.prot5448 (2010)

12. T. Gunther et al., (2015). [*Proc Natl Acad Sci U S A.*](http://www.ncbi.nlm.nih.gov/pubmed/?term=Gunther+et+al+(2015)+atapuerca) **112**. 11917-22 (2015)

13. R. M. Andrews *et al.,* *Nat. Genet.* **23.** 147 (1999)

14. D. M. Behar et al., Am J Hum Genet., **90**. 675-84. (2012)

15. H. Li and R. Durbin, *Bioinformatics* **25**. 1754-60 (2009)

16. H. Li et al., *Bioinformatics* **25**. 2078-9 (2009).

18. Q. Fu *et al.,* *Current Biology* **23**. 553-559 (2013)

17. R. E. Green *et al., EMBO J,* **28**. 2494-502 (2009)

19. R.C. Edgar *BMC Bioinformatics* **5**. 113 (2004)

20. D. Darriba *et al.,* *Nat Methods.* **9**. 772. (2012)

21. R. Bouckaert *et al*., [*PLoS Comput Biol.*](http://www.ncbi.nlm.nih.gov/pubmed/?term=Bouckaert+2014+BEAST)*,* **10**. e1003537 (2014)

22. A. J. Rambaut and A. Drummond, *BMC Evol Biol.* **7**. 214 (2007)

23. J. Krause *et al*., Nature **464**. 894-897 (2010)

24. Q. Fu *et al.,* *Nature* **524**. 216-219 (2015)

25. S. Benezzi *et al., Science* **348**.793-795 (2015).

26. W. Briggs, *Science* **325**. 318-321 (2009).

27. J. Krause *et al*., *Nature* **464**. 894-897 (2010).

28. D. Reich *et al*., *Nature* **468.** 1053-106 (2010).

29. F. Sánchez-Quinto *et al.,* *Current Biology,* **22.** 1494-1499 (2012).

30 C. Gamba *et al*., *Nat Commun.***5**. 5257 (2014).

31 R. Bollongino *et al.,* *Science,* **342.** 479-81 (2013).

32 I. Lazaridis *et al.,* *Nature.* **513**. 409-13 (2014).

33 P. Skoglound *et al*., *Science****.* 344.** 747-50 (2014).

1. A. Seguin Orlando *et al.,* *Science****.* 346.** 1113-8 (2014).

36. W. Haak *et al.,* Nature **522**. 207-11 (2015).

37. Keller *et al.,* Nat Commun **28**. 698 (2012).

38. M. van Oven and M Kayser, *Hum Mutat* **30**:E386-E394 (2009)
